# Augmented Reality–Assisted vs Manual Total Knee Arthroplasty: Clinical and Perioperative Outcomes in a Consecutive Single-Surgeon Cohort

**DOI:** 10.1016/j.artd.2026.102065

**Published:** 2026-06-22

**Authors:** Nathan Farner, Gurkirat Jawanda, Olga J. Santiago-Rivera, Safa S. Kassab

**Affiliations:** a Orthopedic Surgery Residency Program, McLaren Oakland Hospital, Pontiac, MI; b McLaren Health Care, Graduate Medical Education, Grand Blanc, MI; c Orthopedic Specialists of Oakland County, Bloomfield, MI

**Keywords:** Augmented reality–assisted navigation (ARAN), TKA, Total knee arthroplasty, Pixee knee+ augmented reality

## Abstract

**Background:**

Augmented reality–assisted navigation (ARAN) has emerged as an alternative enabling technology for total knee arthroplasty (TKA), providing real-time intraoperative alignment guidance without the capital investment or workflow demands of robotic platforms. Evidence directly comparing ARAN with conventional manual instrumentation on clinical outcomes remains limited.

**Methods:**

We performed a retrospective cohort study of 586 consecutive primary TKAs by a single surgeon between 2022 and 2023. Manual instrumentation (M-TKA) was used in 2022 and ARAN-TKA in 2023. The final cohort included 570 patients. The primary outcome was operative time. Secondary outcomes included 30-day readmissions, opioid utilization in morphine milligram equivalents, and Knee Injury and Osteoarthritis Outcome Score Jr.

**Results:**

Groups were well-matched at baseline. ARAN-TKA was associated with a modest operative time increase (mean difference 3.54 minutes, *P* < .001). Readmissions occurred in 8 M-TKA patients and 4 ARAN-TKA patients, a nonsignificant difference (*P* = .156). Age was the only significant independent predictor of readmission (adjusted odd ratio: 1.12 per year, *P* = .028). Initial opioid prescriptions were lower in the ARAN group (*P* < .001), but 90-day utilization did not differ significantly (*P* = .436). Knee Injury and Osteoarthritis Outcome Score Jr improvements were comparable at 1 year.

**Conclusions:**

ARAN-TKA demonstrated noninferior clinical outcomes and comparable patient-reported improvements vs conventional manual instrumentation. ARAN-TKA was associated with a modest operative time increase, lower initial opioid prescribing not sustained at 90 days, and no significant difference in readmission rates. These findings support the feasibility of integrating ARAN into standard TKA workflows without compromising outcomes. Larger multicenter studies are needed to explore the clinical impact of ARAN-TKA.

## Introduction

Total knee arthroplasty (TKA) is the most common orthopaedic surgery performed, with nearly 700,000 annual cases within the United States alone. [1] Over the past 2 decades, technology has become increasingly integrated into TKA, driven by the goal of improving surgical precision, patient outcomes, and implant longevity. Robotic-assisted surgery has emerged as the prevailing technology-assisted approach, now accounting for 16% of primary TKA procedures in the United States and more than doubling in utilization over the past 5 years. [1] Although robotic systems can improve technical accuracy, these gains have not consistently translated into clinically meaningful improvements in patient-reported outcomes, complications, or satisfaction, and the substantial capital expense, longer operative times, and infrastructure demands of robotic platforms remain significant limitations. [2,3] In response to these limitations, interest has grown in alternative enabling technologies that provide the benefits of intraoperative guidance without the capital investment, infrastructure demands, or workflow disruption associated with robotic platforms. [4]

Augmented reality–assisted navigation (ARAN) has emerged as one such solution. The Pixee Knee + system projects (Pixee Medical, Besancon, France) real-time alignment and resection data directly into the surgeon's field of view via a heads-up display integrated into a camera-equipped surgical headset. Registration is achieved through reusable QR-coded markers pinned within the surgical wound, which are recognized in real time by the headset camera to calculate and overlay mechanical axis alignment and planned resection angles. This eliminates the need for large external arrays, floor-mounted equipment, or additional surgical incisions associated with conventional navigation and robotic systems. [5] Unlike other platforms that require substantial upfront capital investment, the Pixee Knee + ARAN system operates on a per-case utilization model with no capital expenditure, no disposable costs, and no requirement for preoperative advanced imaging, representing a more accessible cost model for institutions evaluating technology-assisted TKA. Early clinical studies of the Knee + system have demonstrated accuracy comparable to robotic-assisted TKA, efficient integration into hospital and ambulatory settings, and low complication rates [[6], [7], [8]] However, limited evidence exists regarding whether ARAN meaningfully influences patient-centered outcomes, operative efficiency, or early recovery when compared directly with conventional manual instrumentation.

The purpose of this study was to evaluate the clinical and perioperative performance of the Pixee Knee + ARAN system compared with conventional manual instrumentation in a consecutive single-surgeon series. We assessed operative time as the primary outcome, with secondary evaluation of 30-day readmission rates, postoperative opioid utilization, and patient-reported outcomes using the Knee Injury and Osteoarthritis Outcome Score Jr (KOOS-JR). We hypothesized that ARAN-assisted TKA would demonstrate noninferior clinical outcomes and comparable operative efficiency when compared with manual instrumentation.

## Methods

This is a retrospective consecutive cohort study. After receiving institutional review board approval, the Michigan Arthroplasty Registry Collaborative Quality Initiative (MARCQI database), hospital, and office medical records were used to retrieve data on TKA surgeries performed by the senior author from the years 2022 to 2023. MARCQI provides prospectively collected, independently abstracted perioperative and outcome data capturing events across the entire state of Michigan, including readmissions and complications managed outside the index institution. Data abstraction is performed by trained independent staff through systematic review of hospital and office records. The data were downloaded by the Principal Investigator and deidentified for analysis purposes.

### Study population

**Figure 1:**
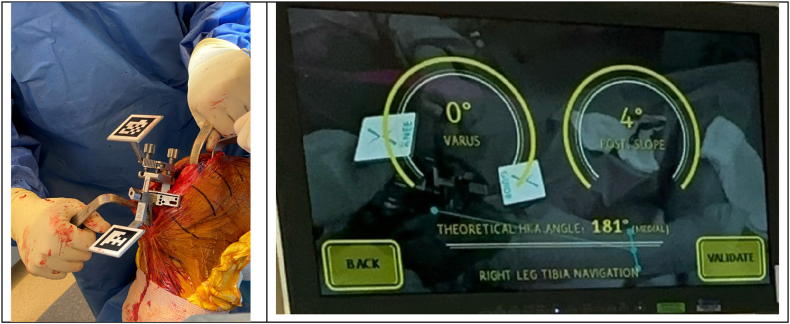
Markers are placed in the wound and are tracked by an external camera worn by the surgeon with a heads-up color display which can be projected to monitors in the operating room.

Our study includes a series of consecutive patients (18 or older) who underwent a primary TKA by a single surgeon at a single institution during 2022 and 2023. All patients in 2022 had TKA performed using manual instrumentation (M-TKA), and all patients in 2023 had TKA performed with the use of ARAN (ARAN-TKA) from Pixee Medical (Pixee Medical, Besancon, France). The Pixee Knee + system projects real-time alignment and resection data into the surgeon's field of view via a heads-up display integrated into surgical glasses equipped with a camera. Registration is achieved through reusable QR-coded markers pinned within the surgical wound, which are recognized in real time by the headset camera. The mechanical axis is determined intraoperatively by rotating the hip to calculate the three-dimensional center of rotation, from which the femoral mechanical and longitudinal axes are derived and overlaid into the surgeon's visual field. The system requires no preoperative calibration, no advanced imaging, and no dedicated system setup prior to patient incision, as all registration and axis calculation are performed intraoperatively. This system has been shown to achieve precision and accuracy comparable to robotic-assisted TKA without the need for intramedullary instrumentation [7,8] Figure 1. All patients had identical techniques except for the use of ARAN. All patients received the Vanguard cruciate-retaining implant (Zimmer Biomet, Warsaw, Indiana). All procedures were performed without the use of tourniquet, with 1 gram dose of prophylactic tranexamic acid and adductor canal block administered by anesthesia. No cases in the ARAN-TKA group required conversion to manual instrumentation. Patients were excluded if they underwent revision TKA, were lost to follow-up, or experienced postoperative trauma. Sixteen patients undergoing a second TKA on the contralateral limb were excluded from the bivariate analysis to ensure statistical independence, yielding a final analytic cohort of 570 patients.

### Measurements

The primary outcome was operative time, measured in 2 intervals: from incision to initiation of closure (“start to close”) and from incision to case end (“start to stop”). Secondary outcomes included 30-day readmission rates, postoperative opioid utilization measured in morphine milligram equivalents (MME), and patient-reported outcomes using the KOOS-JR. The KOOS-JR was measured at presurgery, 2 weeks to 4 months postsurgery, and 1 year. We created a variable that represents the difference between the 2 times (postsurgery minus presurgery). A positive change in score is perceived as beneficial by patients.

### Opioid utilization

Postoperative opioid utilization was quantified in MME using prescription data captured through the Michigan Automated Prescription Monitoring Program (MAPS), the state-mandated controlled substance monitoring database that records all opioid prescriptions dispensed across Michigan for the 90-day postoperative period, regardless of prescribing physician or dispensing pharmacy. Patients with active opioid prescriptions at the time of surgery were excluded from this analysis. Four patients in the M-TKA group with total 90-day MME exceeding 2000 were identified as statistical outliers and excluded from the opioid analysis. MME was analyzed at 2 intervals: the initial postoperative prescription and total cumulative MME prescribed over the 90-day postoperative period.

The main study determinant was the surgical technique defined by the year of surgery. The patients in 2023 had TKA performed with the assistance of augmented reality and those in 2022 had TKA performed using manual instruments. We controlled for baseline-related variables including American Society of Anesthesiologists Physical Status Classification (ASA) score at the time of surgery, body mass index (BMI), sex, and age. These variables were used as adjustment covariates in all regression analyses.

### Data analysis plan

We used frequencies, means, and percentages to describe the study population characteristics. Bivariate analyses including Fisher's exact test, chi-square test, and *t*-test were performed to examine differences between groups (2022 vs 2023). Linear regressions and logistic regressions were used to examine our study outcomes controlling baseline characteristics. As this study included all consecutive primary TKAs performed during the study period, a formal power calculation was not performed. The analytic cohort represents the complete surgical experience of a single surgeon over 2 consecutive years with no exclusions based on sample size. A post hoc power analysis demonstrated an estimated power of approximately 87% at α = 0.05, indicating adequate power to detect the observed difference in operative time.

## Results

A total of 586 consecutive surgeries were completed by a single surgeon during the study period, 262 (45.05%) during 2022 and 322 (54.95%) during 2023. Sixteen cases were excluded from the bivariate analysis due to contralateral TKA performed during the study period. Table 1 presents the characteristics of all cases, including those that were excluded in further analysis (n = 586). Table 2 describes the final study sample (n = 570). There were no significant differences in age, gender, BMI or ASA scores between the 2 intervention groups (age *P* = .312; sex *P* = .794; BMI *P* = .207; ASA *P* = .228; Table 2).

**Table 1:** Description of all patient’s characteristics by type of surgery and by year (N = 586).

Characteristics	Total N = 586	M-TKA (n = 264)	ARAN-TKA (n = 322)
Age (y) Mean (standard deviation)	70.01 (8.32)	69.72 (8.21)	70.24 (8.40)
BMI (kg/m2) Mean (standard deviation)	32.57 (6.72)	32.91 (7.01)	32.33 (6.46)
Number of obese patients (BMI >30 kg/m2)	369 (62.97%)	174 (65.91%)	195 (60.56%)
Number of “Class 3” obese patients (BMI>40 kg/m2)	75 (12.8%)	38 (14.4%)	37 (11.5%)
Sex			
Female	367 (62.6 %)	162 (61.4%)	205 (63.7%)
Male	219 (37.4 %)	102 (38.6%)	117 (36.3%)
ASA score Mean (standard deviation)	2.67 (0.54)	2.70 (0.53)	2.63 (0.55)

**Table 2:** Bivariate association between patient’s characteristics and type of surgery (final study sample n = 570).

Characteristics	Total n = 570	M-TKA (n = 261)	ARAN-TKA (n = 309)	*P* value
Age (y) Mean (standard deviation)	70.05 (8.36)	69.67 (8.25)	70.38 (8.45)	.312^a^
BMI (kg/m2) Mean (standard deviation)	32.65 (6.69)	33.04 (6.94)	32.32 (6.47)	
Number of obese patients (BMI >30 kg/m2)	361 (63.33%)	174 (66.67)	187 (60.52%)	.207
Number of “Class 3” obese patients (BMI>40 kg/m2)	74	38	36	.473
Sex				.794^b^
Female	358 (62.8)	162 (62.1)	196 (63.4)	
Male	212 (37.2)	99 (37.9)	113 (36.6)	
ASA score Mean (standard deviation)				.228^a^
Missing 2 cases 2023	2.66 (0.54)	2.69 (0.51)	2.64 (0.56)	

a *t*-test results.

b Fisher’s exact test.

### Operative time

**Figure 2:**
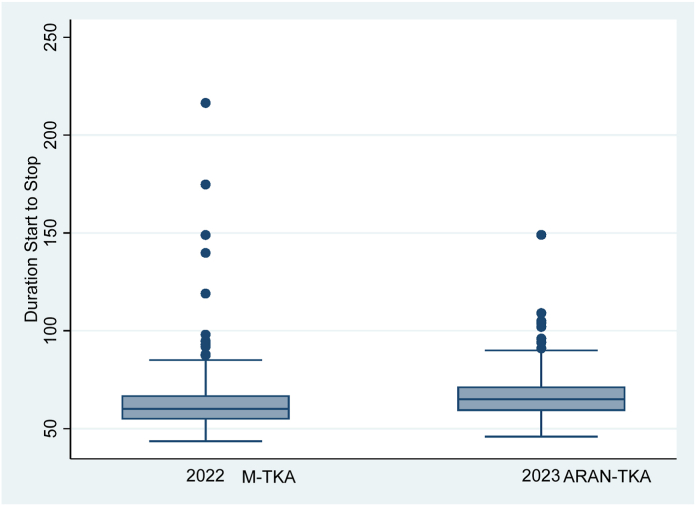
Box-and-whisker plot demonstrates start-to-stop surgical duration, by year and type of surgery.

The mean “start to close” time was 42.99 minutes in the ARAN-TKA group and 39.45 minutes in the M-TKA group (*P* < .001; Table 3). Mean total operative time from “start to stop” was 66.48 minutes for the ARAN-TKA group and 63.00 minutes for the M-TKA group, also reaching statistical significance (*P* = .003; Table 3). The linear regression model sustained the statistically significant differences in operation time at both intervals after controlling for age, sex, BMI, and ASA score (adjusted coefficient: 3.84, 95% confidence interval: 1.99–5.68, *P* < .001; Table 4). Figure 2 demonstrates that both ARAN-TKA and M-TKA have similar operative times with ARAN-TKA showing a slightly higher median (65 vs 59.99 minutes, *P* < .001) and the M-TKA group demonstrating greater variability.

**Table 3:** Bivariate association between study outcomes and type of surgery (n = 570).

Characteristics	Total n = 570	M-TKA (n = 261)	ARAN-TKA (n = 309)	*P* value
Duration (min from “start” to “close”)^c^ Mean (standard deviation)	41.39 (11.89)	39.45 (13.48)	42.99 (10.15)	<.001^a^
Duration (min from “start” to “stop”)^c^ Mean (standard deviation)	64.91 (13.67)	63.0 (16.03)	66.48 (11.09)	.003^a^
30-d readmission (n, %)^b^	12 (2.11%)	8 (3.07%)	4 (1.29%)	.156^b^
Initial postsurgery MME (n = 551) Mean (standard deviation)	218.98 (28.9)	223.45 (24.7)	215.19 (31.6)	<.001
Prescription of MME postsurgery (first 90 d) Mean (standard deviation)	N = 566 292.0 (372.9)	n = 257 293.44 (376.8)	n = 309 290.90 (370.9)	.436^d^
Number of cases with 225Meq	97 (17%)	51 (19.5%)	46 (14.9%)	
Number of cases with no additional MME after surgery	17 (3%)	6 (2.3%)	11 (3.6%)	
Total 90 d MME Mean (standard deviation)	n = 564 497.9 (371.03)	n = 256 503.11 (369.1)	n = 309 493.47 (373.1)	.455^d^
KOOS difference Mean (standard deviation)	n = 468 13.32 (17.4)	n = 207 13.68 (18.7)	n = 261 13.04 (16.5)	.688^a^

a *t*-test results.

b Fisher’s exact test.

c Without 1 outlier, year 2022.

d Nonparametric equality-of-medians test; without outliers n = 4, 2022.

**Table 4:** Individual adjusted linear and logistic regression estimates by study outcomes (n = 570).

Study outcome	Unadjusted coefficient	Adjusted coefficient	SE adjusted	95% CI adjusted	*P* value adjusted
Duration (min from “start” to “close”) (n = 555)	3.84	3.84	.944	1.99, 5.68	<.001
Duration (min from “start” to “stop”) (n = 563)	3.70	3.81	1.09	1.67,5.95	<.001
Prescription of MEq postsurgery (first 90 d) (n = 563)	−3.01	−.265	31.5	−62.2, 61.6	0.993
Total 90 d MEq postsurgery (n = 563)	−10.3	−7.53	32.2	−70.8, 55.7	0.815
KOSS difference (n = 464)	−0.52	−.32	1.63	−3.5, 2.9	0.843

	uOR	aOR	SE adjusted	95% CI adjusted	

30-d readmission (n = 563)	0.40	0.36	.226	0.105, 1.23	0.104

Independent regression analyses. All linear regressions were adjusted by patient’s characteristics, including age, sex, ASA score, and BMI at baseline. All regressions excluded outlier cases of extreme durations, BMI, or MEq prescribed. Significance: *P* < 0.05.

uOR, unadjusted odd ratios; aOR, adjusted odd ratios; CI, confidence interval; SE, standard error.

### Thirty-day readmissions

A total of 12 (2.11%) patients were readmitted within 30 days of surgery, 8 (3.07%) in the M-TKA group and 4 (1.29%) in the ARAN-TKA group. The reasons for readmission are listed in Table 5. This difference did not reach statistical significance (*P* = .156; Table 3). Multivariable logistic regression adjusting for age, sex, BMI, and ASA score identified age as the only significant independent predictor of 30-day readmission (adjusted odd ratio [aOR]: 1.12 per year, 95% CI: 1.04–1.26, *P* = .028). BMI (aOR: 0.95, *P* = .301) and ASA score (aOR: 2.35 per grade, *P* = .343) were not significant independent predictors of readmission. After controlling for all baseline variables, the adjusted odds of readmission remained lower in the ARAN-TKA cohort, though this did not reach statistical significance (aOR: 0.36; 95% CI: 0.105–1.23, *P* = .104; Table 4).

**Table 5:** Thirty-day readmission by surgical group (12 of 570 patients [2.11%]; *P* = .156).

#	Reason for readmission
M-TKA—2022 (8 of 261 patients, 3.07%)	
1	Abdominal pain
2	Documented COVID-19 positive
3	Cecal volvulus/abdominal pain
4	Bleeding duodenal ulcer
5	Atrial fibrillation with rapid ventricular response
6	Altered mental status
7	Delirium/altered mental status
8	Deep vein thrombosis requiring anticoagulation
ARAN-TKA—2023 (4 of 309 patients, 1.29%)	
1	Lower extremity cellulitis
2	New diagnosis of acute myeloid leukemia and respiratory complications
3	Deep vein thrombosis and pulmonary embolism requiring anticoagulation
4	Abdominal ileus

Readmission causes. *P* value from Fisher’s exact test.

M-TKA, manual instrumentation total knee arthroplasty.

### Postoperative opioid utilization (MME)

The mean initial postoperative MME prescription was 223.45 in the M-TKA group and 215.19 in the ARAN-TKA group, representing a mean difference of 8.26 MME that reached statistical significance on bivariate analysis (*P* < .001; Table 3). Total opioid prescribed over the 90-day postoperative period was 503.1 MME in the M-TKA group and 493.5 MME in the ARAN-TKA group, an approximate 10% reduction that did not reach statistical significance (*P* = .436; Table 3). On adjusted linear regression controlling for age, sex, BMI, and ASA score, the initial MME difference did not persist, and neither the initial prescription nor total 90-day MME differed significantly between groups (*P* = .993 and *P* = .815, respectively; Table 4).

### Patient-reported outcome measures (PROM)

**Figure 3:**
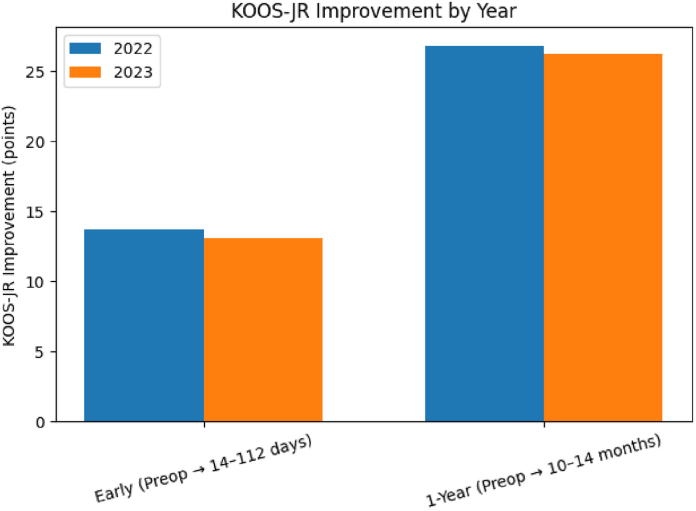
KOOS-JR score difference between 2022 and 2023 with early and late time intervals defined as presurgery to 14-112 days and presurgery to 10-14 months, respectively.

A total of 548 patients completed a preoperative KOOS-JR survey. Baseline scores were comparable between groups (M-TKA 47.64 vs ARAN-TKA 48.73, *P* = .688; Table 3). At early follow-up (14 to 112 days postoperatively), 497 patients completed the KOOS-JR with no statistically significant difference in mean scores between groups (M-TKA 63.00 vs ARAN-TKA 61.51, *P* = .689). At 1-year follow-up (n = 482), both cohorts demonstrated substantial improvement from preoperative baseline, with mean score improvements of 26.8 points in the M-TKA group and 26.2 points in the ARAN-TKA group, and scores remained similar between groups (M-TKA 75.05 vs ARAN-TKA 74.80, *P* = .828; Fig. 3, Table 6).

**Table 6:** Mean scores on the KOOS-Jr. scales by year and type of surgery.

Time interval	2022 (n, mean ± SD)	2023 (n, mean ± SD)	*P* value
Preoperative → 14–112 d	207, 13.69 ± 18.67	261, 13.04 ± 16.46	.689
Preoperative → 10–14 mo	84, 26.78 ± 18.26	149, 26.22 ± 19.40	.828

## Discussion

ARAN has emerged as a promising approach to technology-assisted TKA, with the existing literature demonstrating implant placement accuracy and alignment consistency comparable to robotic-assisted systems while avoiding the capital investment, infrastructure demands, and workflow complexity associated with robotic platforms. [[5], [6], [7], [8]]

However, most existing AR-specific reports have focused primarily on technical accuracy and radiographic alignment rather than patient-centered clinical outcomes and perioperative efficiency. To our knowledge, this represents one of the largest North American consecutive series directly comparing ARAN-assisted TKA with conventional manual instrumentation on clinical and patient-reported outcomes, providing real-world data on the perioperative impact of this technology in a standardized single-surgeon practice.

Introducing technology with TKA has consistently been associated with increased operative time and decreased operating room efficiency. [9,10] The present study demonstrates a mean increase of 3.54 minutes in operative time with ARAN-TKA compared with M-TKA, which was statistically significant (*P* < .001) but is unlikely to be clinically meaningful in routine surgical practice. No intraoperative variables were introduced during the study period except for the adoption of ARAN-TKA in the 2023 cohort, and since this series is from a single surgeon at a single institution, the time difference can reliably be attributed to the change in technique. This represents a marked improvement from the 8-minute increase we reported in our initial series of 17 surgeries utilizing this technology, consistent with that reported by others. [6] Lambrechts et al. demonstrated a 10–14 minute increase in surgical duration across 2 surgeons adopting AR-assisted TKA, with an inflection point in the learning curve at 10–28 cases. [8] Based on this trajectory, it is reasonable to expect that operative times will approach neutrality with continued experience and refinements in surgical workflow.

Importantly, the Pixee Knee + system requires no preoperative calibration, no advanced imaging, and no elaborate system setup prior to patient incision, as all registration and axis calculations are performed intraoperatively. This eliminates the setup and breakdown time associated with robotic platforms [9,11], meaning the 3.54-minute difference observed in this study represents the total additional time attributable to the technology rather than an underestimate of the true workflow impact.

Beyond operative efficiency, the Pixee Knee + ARAN system offers a distinctly different cost model from other technology-assisted platforms. Rather than requiring the substantial upfront capital investment associated with other platforms, the Pixee Knee + operates on a per-case utilization model with no capital expenditure, no disposable costs, and no requirement for preoperative advanced imaging. This pay-per-use structure makes technology-assisted TKA more accessible to a broader range of institutions and ambulatory surgical centers, particularly those unable to justify the infrastructure investment required for robotic platforms.

The 30-day readmission rate was 3.07% in the M-TKA group and 1.29% in the ARAN-TKA group. However, this difference did not reach statistical significance (*P* = .156), and the study was underpowered to detect differences in this low-frequency outcome. Multivariable logistic regression identified age as the only significant independent predictor of 30-day readmission (aOR: 1.12 per year, *P* = .028), with neither surgical technique, BMI, nor ASA score reaching significance, suggesting that readmission risk in this cohort was driven primarily by patient age rather than surgical approach. These results should be interpreted with caution given the limited number of events and require confirmation in larger studies.

Postoperative opioid utilization has been variably reported across technology-assisted TKA studies, and the present findings are consistent with this heterogeneity. [12,13] The less invasive nature of ARAN-TKA, which eliminates intramedullary cannulation and may reduce the need for extensive soft tissue releases, is thought to contribute to lower postoperative pain requirements. While the initial MME prescription was statistically lower in the ARAN-TKA group on bivariate analysis (*P* < .001), this difference did not persist after controlling for baseline patient characteristics in the adjusted regression model (*P* = .993). Total 90-day MME did not differ significantly between groups on either bivariate or adjusted analysis (*P* = .436 and *P* = .815, respectively), indicating that no meaningful difference in postoperative opioid utilization was observed between the 2 surgical techniques. The absence of pain scores in this study limits interpretation of this finding. Larger multicenter studies with prospective pain score collection and standardized opioid protocols will be needed to determine whether technology-assisted TKA produces a clinically meaningful reduction in postoperative opioid requirements.

Patient-reported outcomes using the KOOS-JR were comparable between groups at both early and 1-year follow-up, with no statistically significant differences at any time point. Both cohorts demonstrated substantial and clinically meaningful improvement from preoperative baseline, with mean score improvements at 1 year of 26.8 points in the M-TKA group and 26.2 points in the ARAN-TKA group (*P* = .828). The similarity in PROMs between cohorts is consistent with the emerging AR-specific literature. Panchal et al. recently reported outcomes of 39 consecutive AR-TKAs using the Pixee Knee + system compared with 77 conventional jig-based procedures, demonstrating comparable patient-reported outcomes between groups at 1 year, consistent with the KOOS-JR equivalence observed in the present study. [14] These findings are also consistent with prior literature comparing technology-assisted and conventional TKA. Mostafa et al. reported no meaningful differences in functional scores, satisfaction, or overall PROM trajectories between robotic and manual TKA at short- and mid-term follow-up. [15] Mahoney et al. similarly found no significant difference in postoperative scores between robotic and manual TKA (*P* = .159). [16] While these comparisons reference robotic rather than AR-specific literature, they suggest that technology-assisted systems may optimize technical accuracy and influence certain perioperative metrics without producing meaningful differences in short- to mid-term patient-reported outcomes compared with conventional manual instrumentation.

The present findings should be interpreted within the context of the broader emerging AR-specific literature [14,17,18]. Castellarin et al. reported high implant placement accuracy and improving operative efficiency across a 157-patient single-center AR TKA series, with operative times approaching those of manual instrumentation after an initial learning curve of approximately 20 cases. [7] Lambrechts et al. demonstrated improving operative efficiency with an inflection point at 10–28 cases, consistent with the trajectory observed in the present series. [8] Overschelde et al. reported that greater than 90% of femoral and tibial components achieved alignment within ±3° of the surgical plan using the Pixee Knee + system, with results comparable to robotic-assisted TKA. [18] Panchal et al. further demonstrated that AR-TKA achieved significantly improved femoral coronal alignment accuracy and reproducibility, reduced perioperative blood loss attributed to avoidance of intramedullary canal violation, and improved postoperative range of motion compared with conventional jig-based TKA, with no significant difference in operative time between groups. [14] Taken together, the available AR-specific literature supports the technical accuracy and perioperative safety of ARAN-assisted TKA across different surgical environments and patient populations. The present study contributes to this literature by providing one of the largest North American comparative analyses of clinical and patient-reported outcomes between ARAN and conventional manual instrumentation. Direct comparison of ARAN with other technology-enabling platforms on patient-centered outcomes remains limited and the current study was not designed to address this question. This represents an important direction for future research.

## Limitations

This study has several important limitations. First, as a retrospective single-surgeon, single-institution consecutive cohort study, the results have limited generalizability and may not be applicable to surgeons at different points in their learning curve or to institutions with different patient populations or practice patterns. Second, postoperative opioid utilization was measured through outpatient prescription records via MAPS, representing a proxy for actual consumption, and the absence of postoperative pain scores precluded direct validation of opioid utilization differences between groups. Third, radiographic alignment data were not available, limiting the ability to correlate technical accuracy with clinical outcomes. Fourth, a formal a priori power calculation was not performed, as this study captured all consecutive procedures over a 2-year period. The secondary outcomes including 30-day readmission, MME, and KOOS-JR should be interpreted with caution as the findings are hypothesis-generating rather than definitive.

## Conclusions

ARAN-assisted primary TKA demonstrated noninferior clinical outcomes and comparable patient-reported improvements when compared with conventional manual instrumentation in this consecutive single-surgeon series. ARAN-TKA was associated with a 3.54-minute increase in operative time (*P* < .001), initial opioid prescribing was lower but not sustained at 90 days, and 30-day readmission rates did not differ significantly between groups (*P* = .156). These findings support the feasibility of integrating ARAN into standard TKA workflows, demonstrating that AR guidance can be adopted without compromising operative efficiency or patient outcomes. As adoption of this technology grows, larger multicenter prospective studies are needed to fully explore the clinical, radiographic, and economic impact of ARAN-assisted TKA across diverse surgical environments and patient populations.

## Declaration of generative AI and AI-assisted technologies in the writing process

During the preparation of this work, the authors used Claude (Anthropic) in order to assist with manuscript editing and language refinement. After using this tool/service, the authors reviewed and edited the content as needed and take full responsibility for the content of the published article.

## CRediT authorship contribution statement

**Nathan Farner:** Writing – review & editing, Investigation. **Gurkirat Jawanda:** Writing – review & editing. **Olga J. Santiago-Rivera:** Writing – review & editing, Writing – original draft, Validation, Software, Methodology. **Safa S. Kassab:** Writing – review & editing, Writing – original draft, Investigation.

## Conflicts of interest

S.S. Kassab receives speaking honoraria from Pixee Medical after completion of the study. Pixee Medical had no involvement in the design, data collection, analysis, or manuscript preparation for this study; all other authors declare no potential conflicts of interest.

For full disclosure statements refer to https://doi.org/10.1016/j.artd.2026.102065.

## Appendix A Supplementary data

Conflict of Interest Statement for Jawanda

Conflict of Interest Statement for Kassab

Conflict of Interest Statement for Farner

Conflict of Interest Statement for Santiago-Rivera
