# Supplementary material for: Augmented Reality–Assisted vs Manual Total Knee Arthroplasty: Clinical and Perioperative Outcomes in a Consecutive Single-Surgeon Cohort
Source: Arthroplast Today. 2026 Jun 22;40:102065. doi: 10.1016/j.artd.2026.102065 (PMC13316080; doi:10.1016/j.artd.2026.102065)
Supplement: Conflict of Interest Statement for Kassab [file mmc2.docx]

# CONFLICT OF INTEREST STATEMENT

***American Association of Hip and Knee Surgeons***

(Adopted from the American Academy of Orthopaedic Surgeons disclosure statement)

The following form **must be filled out completely and submitted by each author (example, 6 authors, 6 forms).**

**All items require a response. If there is no relevant disclosure for a given item, enter "*None*.”**

Manuscript Title Comparison of consecutive cohort of adults undergoing total knee arthroplasty with and without the use of augmented reality assisted navigation.

1. Royalties from a company or supplier (The following conflicts were disclosed) None

2. Speakers bureau/paid presentations for a company or supplier (The following conflicts were disclosed) None

3A. Paid employee for a company or supplier (The following conflicts were disclosed) None

3B. Paid consultant for a company or supplier (The following conflicts were disclosed) receiving honoraria from Pixee Medical for speaking and professional activities

3C. Unpaid consultants for a company or supplier (The following conflicts were disclosed) None

4. Stock or stock options in a company or supplier (The following conflicts were disclosed) None

5. Research support from a company or supplier as a Principal Investigator (The following conflicts were disclosed) None

6. Other financial or material support from a company or supplier (The following conflicts were disclosed) None

7. Royalties, financial or material support from publishers (The following conflicts were disclosed) None

8. Medical/Orthopaedic publications editorial/governing board (The following conflicts were disclosed) None

9. Board member/committee appointments for a society (The following conflicts were disclosed) None

**Each author must sign AND print or type his/her name, date and submit a separate form**

In addition, one BLINDED Conflict of Interest form (no author names used) should be submitted per manuscript with all author disclosures.


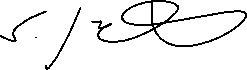


Safa S. Kassab, MD 03/12/2026

Author Name (Print or Type) Author Signature Date
